# Supplementary material for: Domiciliary Carers’ Perspectives on Alcohol Use by Older Adults in Their Care: A Systematic Review and Thematic Synthesis of Qualitative Studies
Source: Int J Environ Res Public Health. 2024 Oct 6;21(10):1324. doi: 10.3390/ijerph21101324 (PMC11506993; doi:10.3390/ijerph21101324)
Supplement: Supplementary file 1 [file ijerph-21-01324-s001.zip › ijerph-3110238-supplementary.pdf]

Supplementary Material

Supplementary Material 1

tiab(age* OR aging OR centenarian OR elder* OR later-life OR "late* life" OR mid-life OR "mid life" OR midlife OR middle-age* OR "middle age*" OR nonagenarian OR octogenarian OR old* OR senescent OR senior* OR sexagenarian OR veteran* OR geriatric OR retir*) AND tiab("home nursing" OR "home care*" OR "home health care*" OR domicil* OR "care work*" OR "social care*" OR "home health aid*" OR "home help*" OR "homemaker service*" OR "cared for" OR "caring for" OR "care of" OR "care for" OR "informal care" OR "family care" NOT "usual care") AND tiab(alcohol* OR drunk OR beverage OR intoxicate OR drink* OR substance* OR "substance use" OR "substance misuse" OR "substance abuse" OR booze OR pissed OR wrecked) AND tiab("semi-structured" OR semistructured OR unstructured OR informal OR "in-depth" OR indepth OR "face-to-face" OR structured OR guide* OR discussion* OR "focus group*" OR qualitative OR ethnograph* OR fieldwork OR "field work" OR "key informant" OR interview* OR "group discussion" OR experience* OR attitude* OR perception* OR attitude* OR belief* OR opinion* OR view* OR perspective* OR voice* OR biograph* OR autobiograph* OR value* OR stor* OR habit* OR choice* OR meaning* OR lifestyle* OR account* OR reason* OR expectation* OR theme* OR "discourse analysis" OR "constant comparative" OR thematic OR narration OR "exploratory research" OR "content analysis" OR transcript* OR "social construction*" OR "grounded theory" or phenomenolog*)

Supplementary Material 2

Reason for Exclusion	Reference
Wrong Population	Allen J, Annells M, Clark E, Lang L, Nunn R, Petrie E, Robins A. 2012. Mixed methods evaluation research for a mental health screening and referral clinical pathway. Worldviews on evidence-based nursing, 9(3), pp. 172-185
	Cabin W. Social workers assert medicare home care ignores social determinants of health. Home Health Care Manag Pract. 2020;32(4):199-205
	Ignaczak CA. Community health nurses' ability to identify and intervene with elderly who are depressed and/or abuse alcohol. United States -- Pennsylvania: University of Pittsburgh; 2001
	Johannessen A, Tevik K, Engedal K, Gade HG, Helvik AS. Health Professionals' Experiences Regarding Alcohol Consumption and Its Relation to Older Care Recipient's Health and Well-Being. Journal of Multidisciplinary Healthcare 2021;14:1829-1842
	Johannessen A, Engedal K, & Helvik AS, Use and misuse of alcohol and psychotropic drugs among older people: Is that an issue when services are planned for and implemented? Scandinavian Journal of Caring Sciences, 2015. 29(2): p. 325-32
	Kellogg FR, Brickner PW. Long-term home health care for the impoverished frail homebound aged: a twenty-seven-year experience. J Am Geriatr Soc. 2000;48(8):1002-1011
	Langdon R, Johnson M, Carroll V, Antonio G. Assessment of the elderly: it's worth covering the risks. Journal of Nursing Management. 2013;21(1):94-105
	Liveng A. The vulnerable elderly's need for recognizing relationships – a challenge to Danish home-based care. Journal of Social Work Practice. 2011;25(3):271-283
	Rodney PA. Toward connectedness and trust: Nurses' enactment of their moral agency within an organizational context. Canada -- British Columbia, CA: The University of British Columbia (Canada); 1997
	Shaw C & Palattiyil G. Issues of alcohol misuse among older people: attitudes and experiences of social work practitioners. Practice: Social Work in Action, 2008. 20(3): p. 181-193
	Wonsil E. Attitudes and Experiences of Palliative Care Providers Toward Patients with Substance Use Disorder. United States -- Washington: University of Washington; 2023

Not Empirical	Counihan CW, Nelson D, Pattullo E. A medicaid mental health carveout program: The massachusetts experience. Manag Care Q. 1996;4(3):85-92
	Gray MT. Habits, rituals, and addiction: an inquiry into substance abuse in older persons. Nursing philosophy : an international journal for healthcare professionals 2014 04;15(2):138-151
	Just MM. Issues in caregiving: elder abuse and substance abuse. Journal of Human Behavior in the Social Environment. 2006;14(1/2):117-137
	Rao T. The role of community nursing in providing integrated care for older people with alcohol misuse. Br J Community Nurs 2014 02;19(2):80-4
	Shanahan PM. Older alcoholics: professional, family education programs aid treatment. Hosp Prog 1984 02;65(2):58-63
Wrong Language	Gunnarsson E. Alkoholmissbruk och sjalvbestammande - en kartlaggning av den svenska hemtjanstens forutsattningar att arbeta med aldre personer med alkoholproblem. Nordic Studies on Alcohol and Drugs 2013;30(3):227-n/a
	Gunnarsson E, Karlsson LB. Äldre, alkohol och omsorgsbehov: Biståndshandläggare om arbetet med äldre personer med missbruksproblem. Nordic Studies on Alcohol and Drugs 2017 02;34(1):43-56
	Menecier P, Moscato A, Fernandez L, Varescon I. Caregivers' representations of elders who drink too much alcohol: crossroads between professional home caregivers and hospitals nurses. Geriatrie et psychologie neuropsychiatrie du vieillissement 2016 Dec 01;14(4):455-463
	Moscato A, Soulas T, Varescon I. Les aidantes professionnelles à domicile dans l'accompagnement de personnes gées atteintes de la maladie d'Alzheimer ou présentant des conduites d'alcoolisation : évaluation qualitative et réflexions cliniques. Canadian Journal on Aging 2016 03;35(1):79-88
Wrong outcome	Johnson M. Addressing Unmet Needs of Female Caregivers of Older Adults: Action Research Study. United States -- Minnesota: Capella University; 2020
	Hokenstad A, Hart A, Gould D, Halper D, Levine C. Closing the home care case: home health aides' perspectives on family caregiving. Home Health Care Manag Pract. 2006;18:306-314
	Sterling MR, Tseng E, Poon A, et al. Experiences of home health care workers in New York city during the coronavirus disease 2019 pandemic: a qualitative analysis. JAMA Intern Med. 2020;180(11):1453-1459
	Salami KK, Okunade OO. Adults and Social Supports for Older Parents in Peri-Urban Ibadan, Nigeria. Journal of Caring Sciences 2020 06;9(2):65-72
Wrong publication type	Oelke ND, Schill K, Szostak C, et al. Supporting the mental health needs of adults 50 and over: The importance of integrated community support services. International Journal of Integrated Care (IJIC). 2016;16(6):1-2
	Galvani, Tetley J, Haigh C, Webb L, Yarwood G, Ashby J, et al. End of life care for people with alcohol and other drug problems: an exploratory study. BMJ Supportive & Palliative Care 2016 09;6(3):392
Wrong study design	Peressini T, McDonald L. An evaluation of a training program on alcoholism and older adults for health care and social service practitioners. Gerontology & Geriatrics Education. 1998;18(4):23-44
